# Cultural competence in dermatology: meeting the needs of Middle Eastern and North African (MENA) and hijabi women

**DOI:** 10.1097/JW9.0000000000000211

**Published:** 2025-06-05

**Authors:** Hanin El-Khateeb, Zaynah Awethe, Abdullah Jalal, Isra El-Khateeb, Mohammed Khatib, Fatimah Jalal, Elizabeth Kiracofe, Susan Massick

**Affiliations:** a Department of Dermatology, The Ohio State University Wexner Medical Center, Columbus, Ohio; b Loyola University Chicago, Loyola University Medical Center, Stritch School of Medicine, Maywood, Illinois; c Oakland University William Beaumont School of Medicine, Rochester, Michigan; d Rosalind Franklin University of Medicine and Science, North Chicago, Illinois; e Campbell University School of Osteopathic Medicine, Lillington, North Carolina; f University of Illinois at Chicago, Chicago, Illinois

**Keywords:** cultural competence, hair thinning, health disparities, hijabi women, Middle Eastern and North African (MENA), patient education

**What is known about this subject in regard to women and their families?**

- Women from the Middle Eastern and North African region and hijabi patients often navigate healthcare systems with a focus on their cultural and religious values.
- They may encounter challenges such as discrimination or bias in medical settings, language barriers, and limited access to female healthcare providers, which can hinder their healthcare experience and outcomes.

**What is new from this article as messages for women and their families?**

- This article provides providers and healthcare workers alike a blueprint to improve communication between healthcare providers and Middle Eastern and North African families.
- It introduces practical measures healthcare systems can adopt to support hijabi patients, such as providing private spaces and modesty-preserving clothing to ensure culturally sensitive and respectful care.

## Dear Editors,

Middle Eastern and North African (MENA) patients are a visible, but often stigmatized group that remains underrepresented in official health records, making them vulnerable in healthcare settings. This paper addresses gaps in culturally competent care for MENA patients, offering recommendations for skin screenings and dermatology visits to enhance healthcare quality.^1^ We also focus on the specific needs of hijabi women, particularly regarding hair thinning and alopecia—issues that are frequently overlooked. While the existing literature acknowledges these needs,^2,3^ our paper provides practical strategies to help dermatologists improve their approach, emphasizing patient education and a comfortable experience.

A 20-question survey conducted at the MAS ICNA Muslim Convention included 80 respondents with an average age of 26 years (Supplementary Table 1, https://links.lww.com/IJWD/A70). Most participants were Arab/Middle Eastern (70%), followed by Southeast Asian (13%), white (5%), and African American (4%). Regarding clinical settings, 71% preferred only the dermatologist present during examinations, and 20% preferred having a family member. The majority (47.5%) favored clinical settings, 18.8% preferred hospital settings, and 0.2% chose health fairs. In addition, 37% preferred a dermatologist of the same ethnicity and 90% preferred the same gender.

Communication preferences varied, with 47% wanting each exam step described as it happened, and 33% preferring a full rundown beforehand. Comfort factors included friendly demeanor (35%), professionalism (26%), and education on findings (22%). In terms of gowns, 28% preferred cloth, while 48% had no preference. Barriers to examinations included perceived lack of need (36%), long wait times (19%), privacy concerns (17.5%), and cost (8%).

The skin concerns reported included acne (31%), dry skin (15%), and hyperpigmentation (10%). Hair concerns were primarily thinning or loss (51%), and scalp issues (16%). While 79% knew that wearing a hijab could lead to hair thinning, only 35% knew protective methods. Regarding knowledge and awareness, 56% relied on the Internet or social media for skin health information and 11% relied on doctors. Despite 60% having seen a dermatologist, 62% did not know how to perform skin self-examination. Sun protection was practiced daily by 32% on hot/sunny days, by 31% when the ultraviolet index was high by 8%, and during prolonged sun exposure by 6%. Only 30% could describe a cancerous lesion morphologically, although 71% were aware of the causes of skin cancer. With regard to protection, 67% cited sunscreen alone, 23% added clothing, and 8% mentioned incorrect methods.

**Figure 1.**
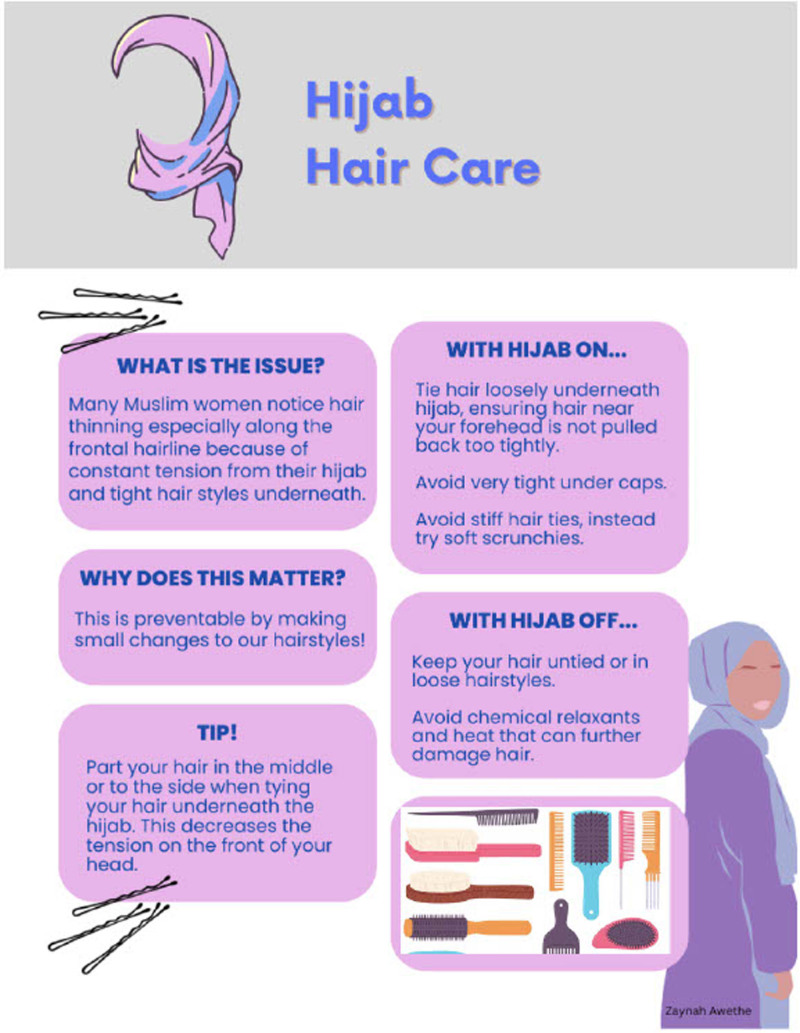
Pamphlet providing tip on hair care for women who observe the hijab. A pamphlet outlining technique to promote healthy hair care while wearing a hijab was aimed at preventing hair thinning and loss. It includes warning signs that may indicate the need to see a dermatologist and provide a QR code linking to the AAD’s “Find a Derm” website for easy access to dermatologists. AAD, American Academy of Dermatology.

Our study highlights the unmet needs of MENA patients and Hijabi women in dermatologic care, emphasizing the importance of cultural sensitivity, and clear communication. Addressing these needs can enhance patient satisfaction and health outcomes. In response to our survey findings that women experienced hair thinning but lacked knowledge of protective methods while wearing the hijab, we created a graphic pamphlet (Fig. 1.) providing best practices for hair health. This initiative underscores the importance of culturally competent dermatology care.

## Conflicts of interest

None.

## Funding

None.

## Study approval

This study was reviewed and approved by BuckIRB; approval #472920. All procedures performed in studies involving human participants were in accordance with the ethical standards of the institutional and/or national research committee and with the 1964 Helsinki Declaration and its later amendments or comparable ethical standards.

## Author contributions

HE-K and ZA: Participated in research design. HE-K, ZA, EK, and SM: Participated in the writing of the paper. HE-K, ZA, AJ, IE-K, MK, and FJ: Participated in the performance of the research. HE-K, ZA, and AJ: Participated in data analysis.

## Supplementary data

Supplementary material associated with this article can be found at http://links.lww.com/IJWD/A70.

## Supplementary Material


