# Supplementary material for: Cultural competence in dermatology: meeting the needs of Middle Eastern and North African (MENA) and hijabi women
Source: Int J Womens Dermatol. 2025 Jun 5;11(2):e211. doi: 10.1097/JW9.0000000000000211 (PMC12140709; doi:10.1097/JW9.0000000000000211)
Supplement: Supplementary file 1 [file jw9-11-e211-s001.pdf]

Supplemental Table 1: This table presents a comprehensive set of questions designed to assess patient comfort, preferences, and barriers related to skin exams.

Question Number	Question
1	Who would you like to be in the room with you during a skin exam?
2	Would you prefer a rundown of all the steps the physician will take during the screening at the start? Or would you prefer having the physician state each step prior to examining a certain area?
3	What could the doctor say that would make you feel comfortable during a skin exam?
4	What environment/room would you feel most comfortable having a full body exam done? (hospital-based setting, clinic, health fair)
5	Do you prefer the examiner to be of the same ethnicity as you? Please indicate how important this is to you. (10 being critically important and 1 being not important at all)
6	What are current or past barriers that have prevented you from receiving a skin exam in the past? (e.g., trust and privacy, negative past experience with a healthcare provider, health beliefs and attitudes, perceived illness and personal health practices, consultancy appointments and waiting time, or communication style/language barriers)
7	During a skin exam, the skin must be exposed to the physician in order to check it appropriately and make sure any concerning lesion is detected. In order to do so, patients are generally asked to remove all of their clothing with the exception of their underwear and then put on a gown. Sometimes the gowns that are used are paper (so that for hygiene reasons they can be thrown away after the exam), and sometimes they are made of cloth. How would you feel the most comfortable?
8	Do you prefer the examiner to be of the same gender as you?
9	What are some questions or comments the physician could say that would make you feel comfortable during an exam?

10	What are current or past barriers that have prevented you from receiving a skin exam in the past?
11	What are some of your concerns regarding your skin?
12	Do you know how to check your skin for abnormalities?
13	Have you seen a dermatologist in the past?
14	What happens to your skin in the sun?
15	How do you decide when to take sun protection measures?
16	What adjectives come to mind when you think of skin cancer?
17	What do you use to protect your skin from the sun or harmful radiation?
18	Where do you get information about sun protection and skin cancer?
19	What do you perceive your ethnicity as?
20	Are you aware of what may cause skin cancer, and how to protect yourself?
21	Did you know that the wearing of a hijab can be associated with hair loss?
22	Did you know there are ways to wear a hijab in order to better protect against hair loss and thinning?
23	What are some of your concerns regarding your hair?
24	What are current or past barriers that have prevented you from visiting a dermatologist for your hair?
